# The landscape of mitochondrial small non-coding RNAs in the PGCs of male mice, spermatogonia, gametes and in zygotes

**DOI:** 10.1186/s12864-018-5020-3

**Published:** 2018-08-28

**Authors:** Eduardo Larriba, Eduardo Rial, Jesús del Mazo

**Affiliations:** 1 Department of Cellular & Molecular Biology, Centro de Investigaciones Biológicas C.I.B. (CSIC), Ramiro de Maeztu 9, 28040 Madrid, Spain; 2 Department of Chemical & Physical Biology, Centro de Investigaciones Biológicas C.I.B. (CSIC), Ramiro de Maeztu 9, 28040 Madrid, Spain

**Keywords:** Small non-coding RNA, Mitochondria, Primordial germ cells, Gametes, Zygotes

## Abstract

**Background:**

Mitochondria are organelles that fulfill a fundamental role in cell bioenergetics, as well as in other processes like cell signaling and death. Small non-coding RNAs (sncRNA) are now being considered as pivotal post-transcriptional regulators, widening the landscape of their diversity and functions. In mammalian cells, small RNAs encoded by the mitochondrial genome, mitosRNAs were discovered recently, although their biological role remains uncertain.

**Results:**

Here, using specific bioinformatics analyses, we have defined the diversity of mitosRNAs present in early differentiated germ cells of male mice (PGCs and spermatogonia), and in the gametes of both sexes and in zygotes. We found strong transcription of mitosRNAs relative to the size of the mtDNA, and classifying these mitosRNAs into different functional sncRNA groups highlighted the predominance of Piwi-interacting RNAs (piRNAs) relative to the other types of mitosRNAs. Mito-piRNAs were more abundant in oocytes and zygotes, where mitochondria fulfill key roles in fecundation process. Functional analysis of some particular mito-piRNAs (mito-piR-7,456,245), also expressed in 3T3-L1 cells, was assessed after exposure to RNA antagonists.

**Conclusions:**

As far as we are aware, this is the first integrated analysis of sncRNAs encoded by mtDNA in germ cells and zygotes. The data obtained suggesting that mitosRNAs fulfill key roles in gamete differentiation and fertilization.

**Electronic supplementary material:**

The online version of this article (10.1186/s12864-018-5020-3) contains supplementary material, which is available to authorized users.

## Background

Mammalian mitochondria are organelles that are thought to have an originated through endosymbiosis of α-proteobacteria [1]. Their role in oxidative phosphorylation (OXPHOS) has led the mitochondria to be considered as the “powerhouse” of the cell, although they have also been implicated in the regulation of many other processes, including: apoptosis, calcium homeostasis, aging, signaling, stem cell renewal and immune responses [2, 3]. Consequently, mutations or alterations that affect mitochondrial gene expression may condition a wide range of diseases [4] (http://www.mitomap.org/MITOMAP), including different types of cancers [5]. No curative treatments are currently available for disorders provoked by mitochondrial DNA (mtDNA) [3], with the particular exception of mitochondria replacement therapies in oocytes and zygotes [6].

In mammals, some mitochondrial genes are contained in specific coding regions of the nuclear DNA but the majority are found in compact circular DNA (1.6 Kb) inside the mitochondria (mtDNA). This mtDNA encodes 37 genes (2 rRNAs, 22 tRNAs and 13 protein coding genes that are part of the OXPHOS system [7]) and it has a regulatory non-coding region that contains two promoters (PH and PL), the D-Loop [8]. Mitochondrial genes are asymmetrical distributed in the two mtDNA strands and they are transcribed from the PH and PL promoters as a long polycistronic precursor [8]. Moreover, the D-loop region contains the origin of replication for the heavy (or H) chain, whereas the origin of replication of the light (or L) chain is displaced by approximately two-thirds of the mtDNA, within a cluster of five tRNAs [8]. Interestingly, there is a coordinated switch between the transcription and replication of mtDNA, which takes place in this D-loop region [9].

Non-coding RNAs (ncRNAs) are key post-transcriptional regulators of gene expression in normal and pathological processes of cell differentiation and development [10–12]. There are two main types of ncRNAs in mammalian cells, small (18–35 nts, sncRNAs) and long ncRNAs (lncRNAs), the former including microRNAs (miRNAs), Piwi-interacting RNAs (piRNAs) and endogenous-small interfering RNA (endo-siRNAs), as well as other classes of sncRNAs derived from tRNAs, rRNAs and small nucleolar RNAs (snoRNAs) [10, 13]. Recent next generation sequencing (NGS) studies identified different types of sncRNAs that are associated with the mitochondrial genome of mammalian cells, such as miRNAs [14–16]. These sncRNAs produced from the mitochondrial genome were proposed to interact with and regulate different pathways that communicate it with the nuclear genome [17], and thus, mitochondrial-associated RNAs could play an important role in pathways related to cell synchronization [17]. These findings led to the definition of a new sub-group of sncRNAs encoded by the mitochondrial genome: mitosRNAs. In fact, studies of mitochondrial miRNAs in different species have established the idea that miRNAs and the RISC machinery interact in the mitochondria, designating these as mito-miRNAs [16, 17].

PiRNAs correspond to a class of sncRNAs that were discovered in germline cells and classically, they have been associated with “genome defense” against transposable elements (TEs) [18, 19]. However, the role of piRNAs seems not to be restricted to germline cells and in somatic cells, piRNAs participate in epigenetic reprogramming and the regulation of gene expression [18, 20]. PiRNA sequences produced by the mitochondrial genome have since been identified [21], although there is still no evidence of piRNAs or PIWI proteins in the mitochondria of mouse cells to date. However, PIWIL-1, a human homolog of the mouse MIWI protein, has been detected in the mitochondria of human cancer cells [21].

A recent NGS analysis of mouse sncRNAs in male and female gametes, and in zygotes, highlighted the diversity of sncRNAs in these cell types, suggesting they might fulfil crucial roles in gamete differentiation and fertilization [19, 22]. Having generated a specific bioinformatics workflow to analyze the sequences from these mouse libraries in greater depth, we have identified a more extensive population of mitosRNAs in early male germ cells (primordial germ cells –PGCs- and spermatogonia), in both gametes (oocytes and spermatozoa) and in zygotes. To assess the possible function of some of these specific mito-piRNAs, we evaluated the differentially expression of mitosRNAs detected in germ cells or zygotes from our previous databases of piRNAs, some of which were also present in a somatic mouse cell line, 3T3-L1 cells. As such, molecular, cellular and physiological approaches were used to evaluate the potential role of these specific mitosRNAs in murine cells. 

## Results

### Identification of sncRNAs associated with mitochondria in germ cells and the zygote

**Fig. 1:**
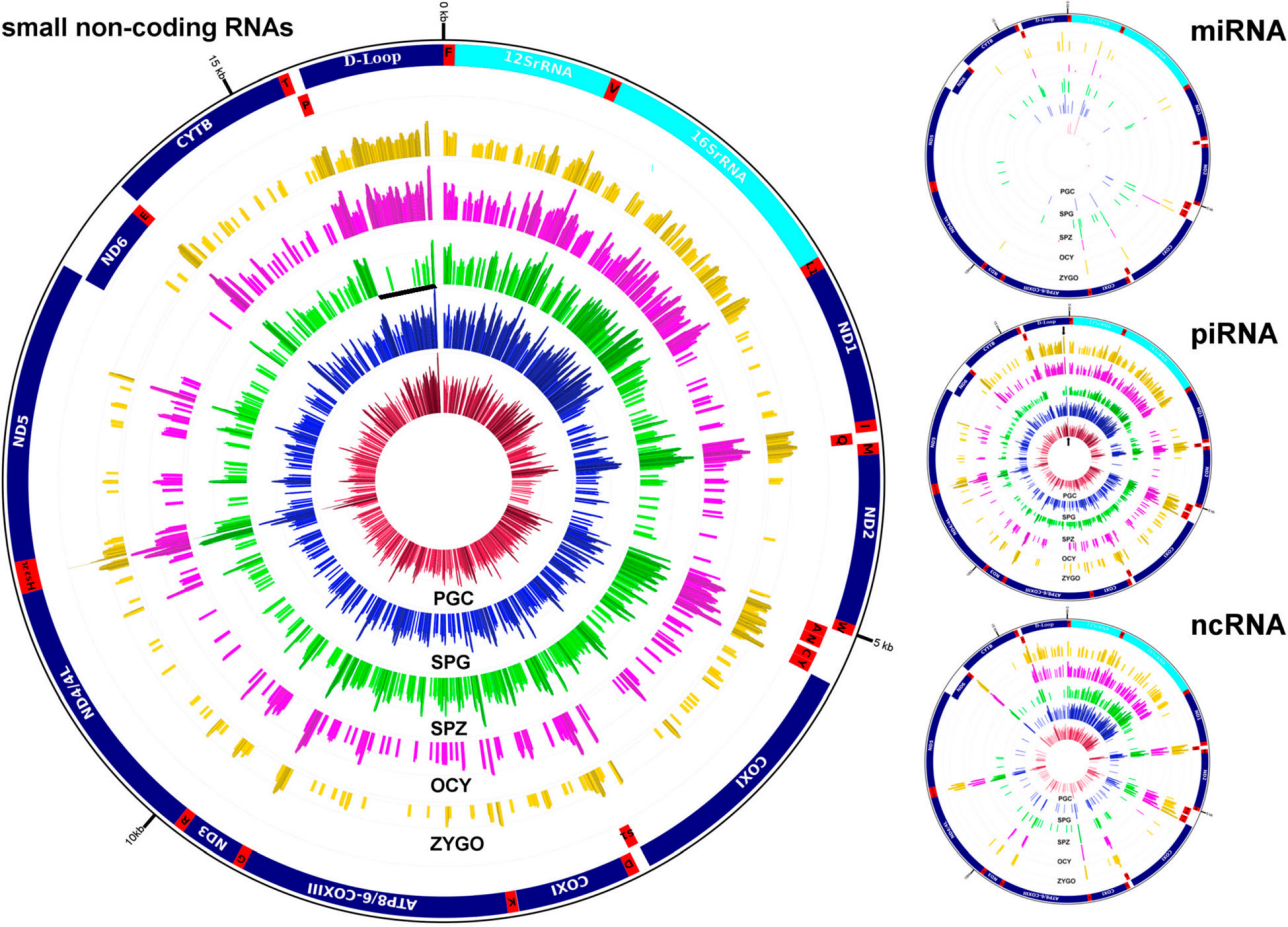
The sncRNAs sequence coverages in the mitochondrial genome of male PGCs, spermatogonia, spermatozoa, oocytes and zygotes. Circular representation of the sncRNA coverage in mouse mtDNA. The radial bars represent the log transformation of the sncRNA read coverage. Reads from: primordial germ *cells* (PGC) are represented in red; spermatogonia (SPG) in blue; spermatozoa (SPZ) in green; oocytes (OCY) in purple; and zygotes (ZYGO) in gold. Annotation of the mitochondrial genes (dark blue), and the rRNA (light blue) and tRNA (red) was obtained from the Ensembl database. The read coverage was obtained using BedTools software and the circular representation was created using the Circleator tool. Coverage of the piR-7,456,245 region is indicated in the piRNA circle by a black arrow in the corresponding piRNA circle

NGS analyses of the sncRNAs in mouse male PGCs at E13, spermatogonia cells (SPG), spermatozoa (SPZ), oocytes (OCY) and zygotes (ZYGO) suggested important regulatory roles of these sncRNAs in fertilization and gamete differentiation [13, 22]. These NGS libraries could be used to identify the sncRNAs associated with the mouse mitochondrial genome (mitosRNAs) following a specific bioinformatics pipeline (Additional file 1: Figure S1), an approach that identified more than 250,000 sncRNA reads associated to the mitochondrial genome in all the cell types studied (Table 1). Although these mitochondrial sncRNAs reads represent less than 1% of the total sncRNA reads, we found that the mitochondria in the mouse cells analyzed were enriched in sncRNA molecules (Additional file 1: Figure S2), with the exception of the SPZ (Table 1), particularly when taking into account the amount of mouse mtDNA (Kbs/sncRNA reads) relative to the nuclear DNA. In general, all mitochondrial genes encoded mitosRNAs, including non-coding rRNAs, tRNAs and the D-loop region (Fig. 1), although cell specific patterns of mitosRNA production from mtDNA was evident among the cell types analyzed (Fig. 1). Hence, the regulation of mitosRNAs expression would appear to be cell type specific. This intense production of mitosRNAs from mtDNA and their specific cell patterns suggest potentially important roles for these sncRNAs in mitochondrial regulation.

**Table 1:** Global analysis of the small RNA population associated with mouse mitochondria

	PGC	SPG	SPZ	OCY	ZYGO
Total reads in NGS libraries	39,792,571	39,531,404	28,519,791	50,561,193	49,555,453
Reads associated with MT	366,447	258,125	346,020	281,005	338,956
% of reads associated with MT	0.92	0.65	1.2	0.55	0.68
Total sequences in NGS libraries	567,704	831,190	324,746	457,580	454,901
Sequences associated with MT	4859	6393	5496	3702	3399
% of sequences associated with MT	0.8	0.7	1.7	0.8	0.7

### Classification of mitochondrial sncRNAs

**Fig. 2:**
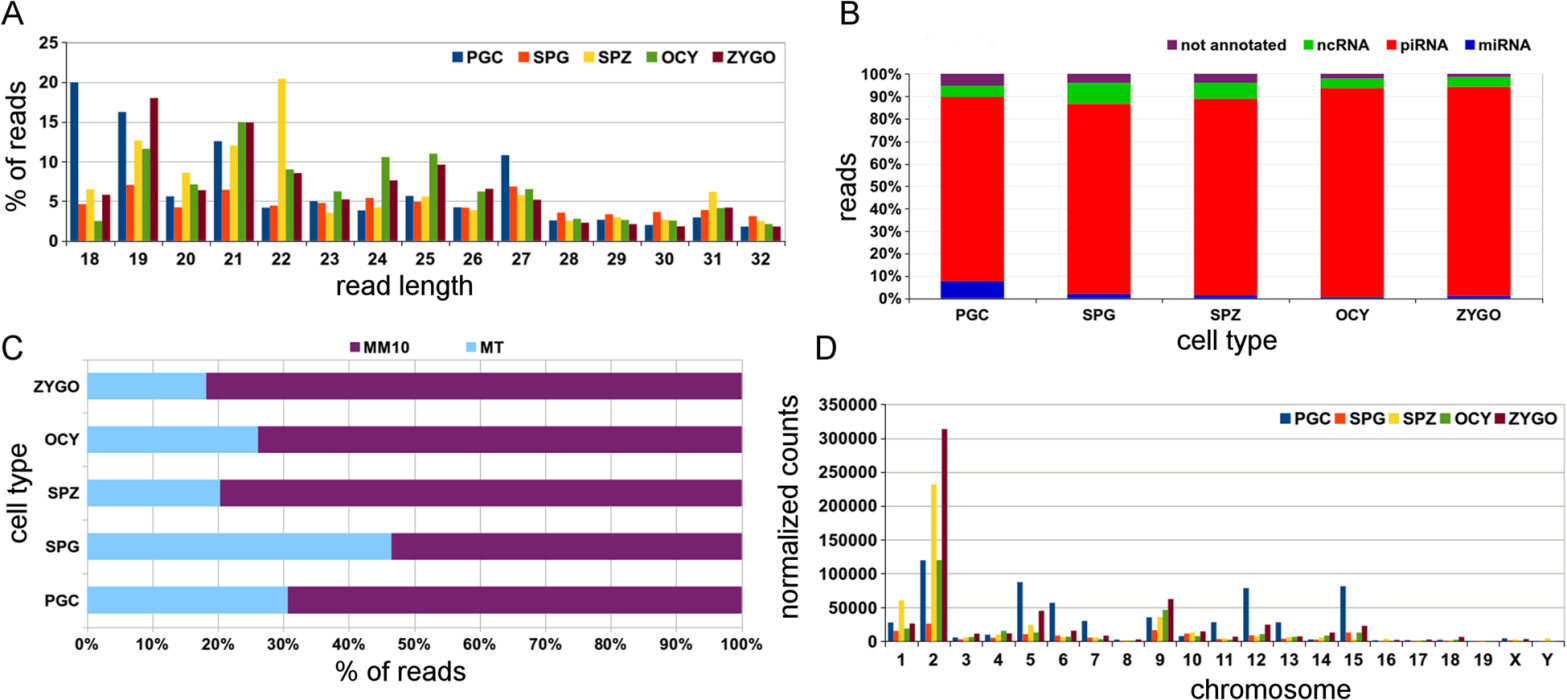
Characterization of the mitochondrial sncRNA populations in male PGCs, spermatogonia and spermatozoa, and in oocytes and zygotes. **a** Read length distribution of mitochondrial derived sncRNAs from different cell types. The percentage of reads was calculated from the total reads in the small RNA-Seq library. **b** Classification of mitosRNAs in microRNAs (miRNA - mito-miRNAs), PIWI-interacting RNAs (piRNAs - mito-piRNAs) and sequences from non-coding RNAs present in the Ensembl database (ncRNAs). Reads that do not map to previous databases are considered as not annotated. **c** The percentage of mitochondrial encoded sncRNAs that map to the mouse genome (MM10 - including nuclear mitochondrial sequences) and those exclusive to mitochondrial DNA (MT). **d** Chromosome distribution of the mitosRNAs derived from the mouse genome. Normalization of the read count was carried out using DESeq. Male cells: primordial germ *cells* (PGCs), spermatogonia cells (SPG), and spermatozoa (SPZ). Female cells: oocytes (OCY) and zygotes (ZYGO)

The sncRNA populations were essentially composed of miRNAs (~ 20–24 nucleotides), piRNAs (~ 24–31 nucleotides) and other small RNAs derived from ncRNAs like tRNAs, rRNAs and snoRNAs [10]. We performed a read length analyses of the mitosRNAs identified (Fig. 2a) and the patterns of sequence lengths suggested the mitosRNAs represent a distinct population of sncRNAs. We used a bioinformatics pipeline that incorporated information from different sncRNA databases to classify these mitosRNAs, which classified 80 to 90% of the mitosRNA reads as piRNAs (Fig. 2b, Table 2), particularly in the OCY and ZYGO (Fig. 2b, Table 2). Interestingly, 8% of the mitosRNA population in PGC cells were miRNAs, which were less predominant in the other cell types and they represented as little as 0.6% in OCY (Fig. 2B, Table 2).

**Table 2:** Classification of sncRNAs associated with mtDNA

	miRNAs		piRNAs		sncRNAs	
Cell type	Reads	Sequences	Reads	Sequences	Reads	Sequences
PGC	27,979	56	300,654	3135	18,411	708
SPG	5204	38	218,287	4403	24,241	1178
SPZ	4133	43	303,487	4152	24,653	911
OCY	1766	25	261,366	2703	12,004	553
ZYGO	3558	38	315,773	2502	14,679	530

Another interesting issue is whether these mitosRNAs may be derived from the nuclear DNA. To address this question, mitosRNA reads were mapped against the mouse nuclear genome (mm10). We detected that more than 70% of the mitosRNAs mapped again mouse nuclear DNA (Fig. 2c) in gametes and zygotes, reaching a maximum in zygotes where they represented 81% of the mitosRNA reads. Analyzing the chromosome distribution of the mitosRNA indicated an enrichment on chromosome 2 for all cell types (Fig. 2d) and it was noteworthy that mitosRNAs from PGCs had a distinct chromosome profile than in the other cell types (Fig. 2d). Moreover, the analysis of specific nuclear mitochondria sequences (NUMTs) in the mouse nuclear genome indicated that the distribution of the mitosRNA sequences was not biased to the NUMTs (Additional file 1: Figure S3). In mammals, the insertion of NUMTs is not random and it is associated with regions enriched in TEs [23–27]. These results were consistent with the hypothesis proposing mitosRNAs as key elements in the communication between mitochondria and the nucleus [17].

### Analyses of mitochondrial-associated miRNAs

MicroRNAs are post-transcriptional regulators of mRNA expression and their activity is driven by Argonaute (AGO) proteins [24]. Different studies detected AGO2 in mitochondria purified from human and mouse cells or tissues, indicating that miRNAs and RISC may be active in mitochondria [25, 26]. Furthermore, the presence of miRNAs inside the mitochondria of different cell types, tissues and species is strong evidence that mito-miRNAs are more than just a potential sequencing artifact [26]. In all cell types examined, the miRNA populations associated to mtDNA were smaller than those of other small non-coding populations (piRNAs: Table 2). Interestingly, this difference in the number of annotated reads in PGCs is more than one order of magnitude that of other samples for miRNAs. However, the diversity of the miRNAs, does not presented the same increase (Table 2).

**Fig. 3:**
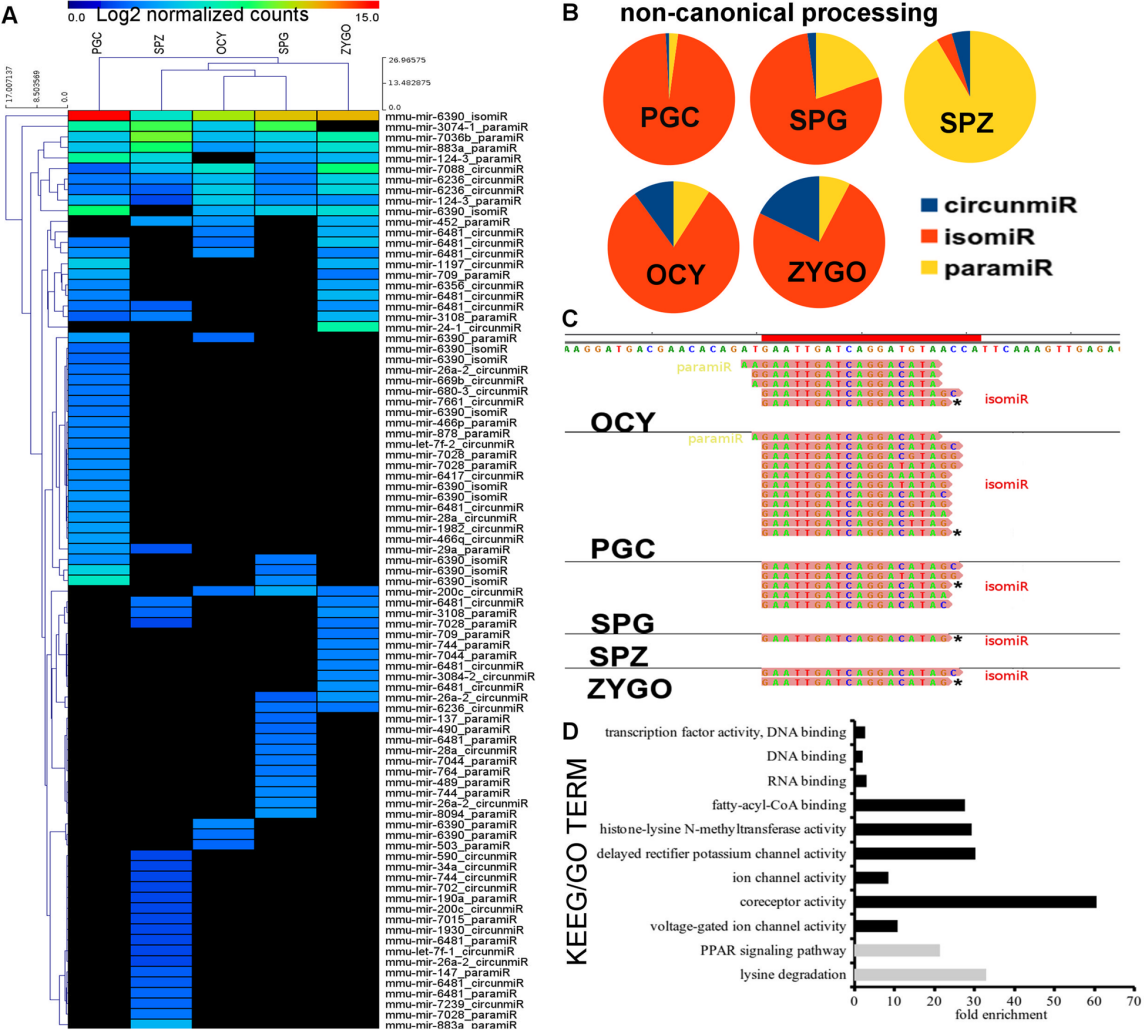
Analysis of the miRNAs derived from mtDNA in male PGCs, spermatogonia, spermatozoa, and in oocytes and zygotes. **a** Non-supervised hierarchical clustering of miRNA and miRNA isoform counts associated with the mitochondrial genome in the different samples. Read counts were normalized using DESeq and transformed to a base two logarithm. The annotation corresponds to the pre-microRNA and the miRNA isoforms. **b** Distribution of the different isoforms of miRNAs derived from mtDNA. MicroRNA isoforms: isomiR, miRNA isoforms with the same seed sequence as canonical and trimmed nucleotides in the 3′ region; paramiR, miRNA isoform where the 5′ region corresponds to the canonical miRNA sequences; and circumiR, the miRNA isoform where the 3′ region corresponds to the canonical miRNA sequences. **c** Different isomiRs and paramiRs found in *miR-6390* in the different cell types: red bar corresponds to the canonical form of the *miR-6390*; the asterisks indicate the most expressed miRNA isoform in each sample. **d** Enrichment of KEEG pathways among the *miR-6390* targets. Male cells: primordial germ *cells* (PGCs), spermatogonia cells (SPG), and spermatozoa (SPZ). Female cells: oocytes (OCY) and zygotes (ZYGO)

The microRNA isoforms differ from the canonical sequences due to variations at the 3`- and/or the 5`-end(s) [24]. We classified the mito-miRNA isoforms according to the miRNA isoform present: 3′ deletions of canonical miRNA sequences (isomiRs); partial correspondence with the 5′ region of the canonical miRNA (paramiR); and partial correspondence with the 3′ region of the canonical miRNA sequences (circumiR). All the miRNAs encoded by mtDNA were isoforms of canonical miRNAs (Fig. 3a, b). A hierarchical clustering of the mito-miRNA isoforms reflected specific cell expression patterns (Fig. 3a). We found that the expression pattern of mito-miRNA isoforms was more similar in OCY and SPG than in SPZ and ZYGO (Fig. 3a), and that there was a greater diversity of mito-miRNA isoforms in PGC cells (Fig. 3b). Interestingly, more than 90% of the mito-miRNA isoforms in SPZ were classified as paramiRs (Fig. 3b), mito-miRNAs that retain a partial correspondence with the 5′ region of the canonical miRNAs. Different isoforms of mito-miRNAs encoded in the mtDNA displayed cell specific expression and diversity, suggesting a possible function that remains as yet unknown.

### The mitochondrial-associated mmu-miR-6390 isoform

Despite the distinct expression of the mito-miRNA isoforms in different cell types, we identified a specific mito-miRNA isoform expressed in all samples, isomiR-6390 (Fig. 3a). Although there are different isoforms of miRNA-6390, which are usually isomiRs and paramiRs (Fig. 3c), the isomiR-6390 sequence was detected in all the cell types studied (Fig. 3c). The target genes of these *miR-6390* isoforms have the same seed sequence as the canonical *miR-6390* (Fig. 3c). Thus, we identified 46 potential targets of the predicted miRNA:target interactions in two databases (Additional file 2: Table S2) and the functional annotation of these isomiR-6390 targets highlighted an enrichment in activities implicated in DNA and RNA binding, histone modification and the β-oxidation pathway (Fig. 3d). Remarkably, we found enrichment in KEGG pathways related to lysine degradation and the peroxisome proliferator-activated receptors (PPAR) signaling pathway associated to the metabolism of fatty acids and their derivatives (Fig. 3d). Interestingly, PPAR signaling pathways participate in the regulation of mitochondrial β-oxidation [26].

### Expression of Mito-piRNAs

**Fig. 4:**
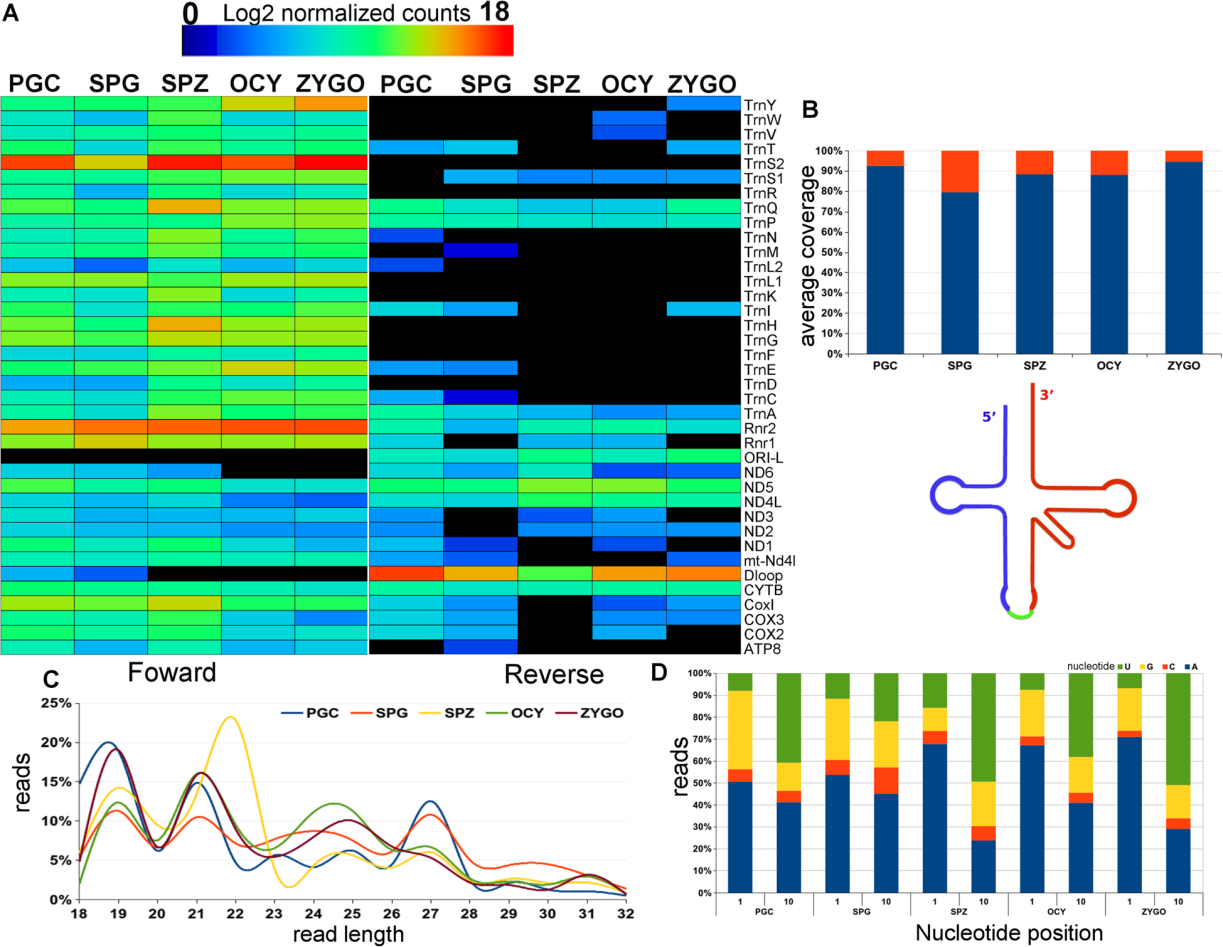
Expression of mitochondrially encoded piRNAs and hallmarks of the mitochondrial piRNA populations from male PGCs, spermatogonia and spermatozoa, and oocytes and zygotes. **a** Expression heatmap of piRNAs generated from different mitochondrial genes where the heatmap color indicates the normalized expression of piRNAs derived from mitochondrial genes. The annotation of mitochondrial genes was obtained from Ensembl database. **b** Coverage distribution of the piRNAs from tRNAs in different samples, the graphs representing the median coverage of piRNA reads in the different tRNA samples. Schematic representation of tRNA arms. **c** The read length distribution of the piRNA populations derived from the mtDNA. The read percentage was calculated using the total number of piRNA reads in each sample. **d** Nucleotide frequency at the first and tenth nucleotide of the piRNA reads. Male cells: primordial germ *cells* (PGCs), spermatogonia cells (SPG), and spermatozoa (SPZ). Female cells: oocytes (OCY) and zygotes (ZYGO)

While piRNAs have classically been associated with protection against TEs in germ cells [19, 27], new biological roles of piRNAs are now being identified [18, 20]. Indeed, piRNA and PIWI proteins have been found in the mitochondria of human [21] but not in mouse cells, and to our knowledge not in mammalian PGCs, spermatogonia, gametes of either sex or zygotes. Interestingly, mito-piRNAs were the most predominant mitosRNA population in the mitochondria of these cell types (Fig. 2c, Table 2). Also, all regions of the mouse mitochondrial genome encoded mito-piRNAs, and both mtDNA strands (Figs. 1 and 4a). The strongest mito-piRNA expression corresponded to the forward strand of mitochondrial tRNAs (mt-tRNA) and 16S rRNA (Fig. 4a). However, strong expression of mito-piRNAs was detected from the mtDNA reverse strand in the non-coding region of the D-loop (Figs. 1 and 4a). Remarkably, sperm cells had the lowest mito-piRNA coverage in the D-loop region with respect to the other cell types (Fig. 1, black line in the SPZ circle).

More than 80% of the mito-piRNAs were derived from 5′ mt-tRNA arms in all cell types (Fig. 4b, Additional file 1: Figure S4), and they were more predominant in the PGC and ZYGO samples (Fig. 4b, Additional file 1: Figure S4). The sequence length distribution of mito-piRNAs (Fig. 4c) showed a predominant peak of small (19 and 21 nt) piRNA sequences in all samples (Fig. 4c), possibly related to the unusually small RNAs (usRNAs) [28] and endo-siRNA sncRNAs, respectively [13, 22]. However, there was another predominant peak of 25 nt piRNAs from OCY and ZYGO (Fig. 4c), and in PGCs and spermatogonia at 27 nt (Fig. 4c). The different sizes of these mito-piRNA populations has been associated with the selective interaction of murine PIWI proteins with piRNAs of different lengths [18, 29]. We performed an analysis of the nucleotide frequency at position 1 and 10 of the mitochondria encoded piRNAs (Fig. 4d), demonstrating that mito-piRNAs did not present a bias (Fig. 4d) for uridine as the first 5′ nt (1 U) or for adenine at position 10 (10A), a hallmark of primary and secondary nuclear piRNAs, respectively [18, 22]. Finally, the human PIWIL1 protein (MIWI in mouse) was detected in the mitochondria of human cancer cells [21] but not in mouse mitochondria. Using an in silico approach, we identified an isoform of the MIWI2 protein (PIWIL4–201, Ensembl transcript name) that presented a high probability of localizing to the mitochondria (Additional file 2: Table S3). These results represent the first description and characterization of mitochondrial encoded piRNAs in male mouse germ cells, and in gametes of both sexes and in zygotes. 

### Mito-piRNA function

To address the potential functions of mito-piRNAs, we applied our bioinformatics pipeline to three small-RNA sequence datasets from 3T3-L1 fibroblasts available in the GEO database (Additional file 2: Table S4). We identified 15,881 to 51,290 (average 33,156) reads from 3T3-L1 mtDNA associated NGS libraries (Additional file 2: Table S4). Interestingly, two of the 3T3-L1 libraries analyzed were enriched in 29 nt reads associated with mtDNA (Additional file 1: Figure S5A) and we found that mito-piRNAs were the most highly represented mitosRNAs in all three libraries (Additional file 1: Figure S5B). There was a variation in the number of mitosRNAs that map to nuclear DNA in the 3 libraries, which was 50% on average (Additional file 1: Figure S5C). Interestingly, mitoRNAs from 3T3-L1 cells were also enriched on chromosome 2 (Additional file 1: Figure S5D).

**Fig. 5:**
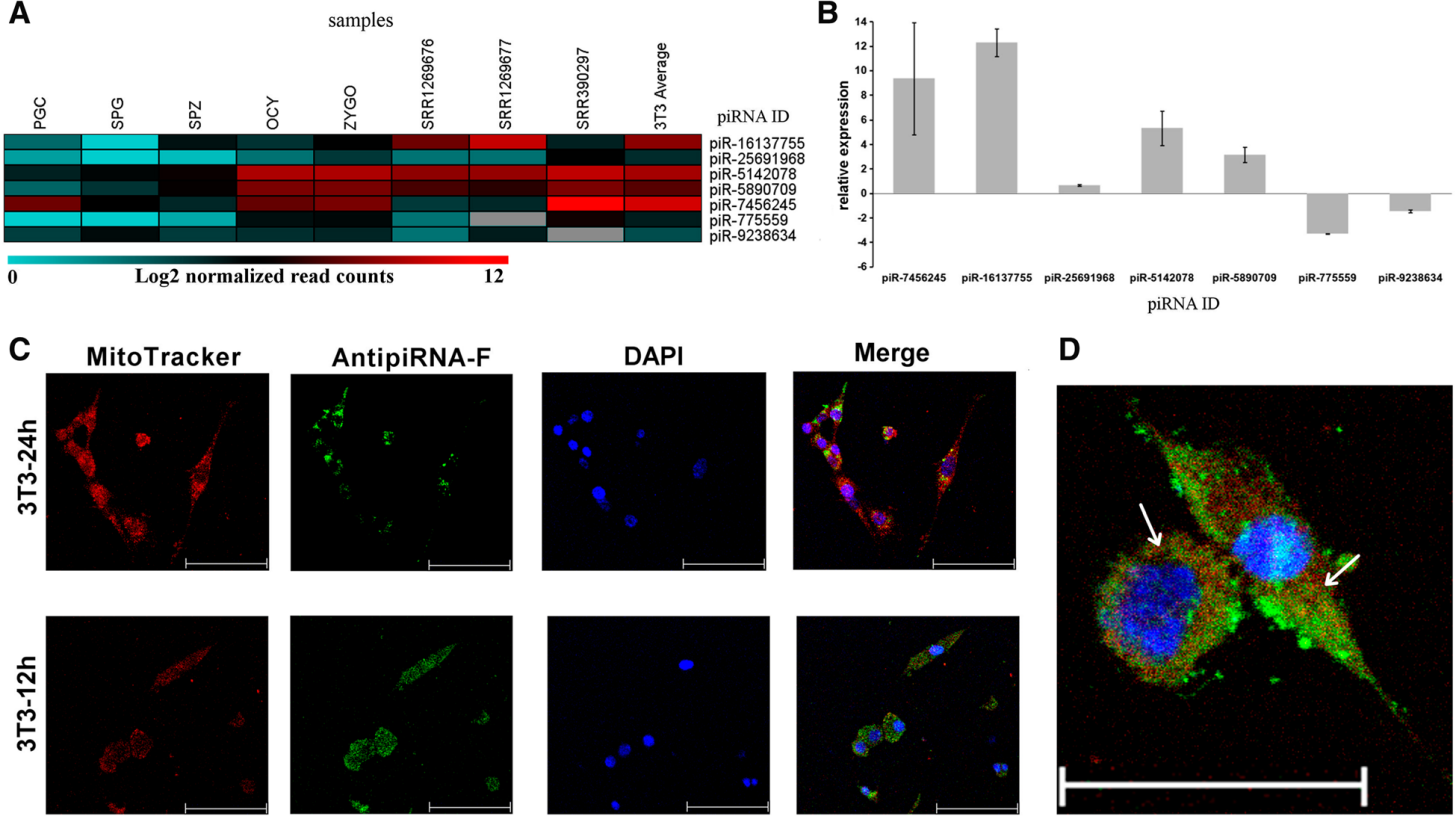
Quantification and analysis of the *mito-piR-7,456,245* derived from mitochondria in 3T3-L1 cells. **a** Expression of 7 selected piRNAs in NGS libraries. The heat map colors correspond to log_2_ of the normalized read counts. **b** The RT-qPCR expression of 7 selected piRNAs in arbitrary units calculated using U6 derived piRNA as an endogenous standard. **c** Confocal images of cultured 3T3-L1 cells transfected with AntipiRNA-F fluorescent GapmeR, and stained with MitoTracker (mitochondria) and DAPI (nucleus). **d** Merged image of 3T3-L1 cells transfected with AntipiRNA-F fluorescent GapmeR, and stained with MitoTracker and DAPI. The arrows indicate the partial localization of the AntipiRNA-F fluorescent GapmeR and the mitochondria. Male cells: primordial germ *cells* (PGCs), spermatogonia cells (SPG), and spermatozoa (SPZ); female cells: oocytes (OCY); and zygotes (ZYGO). Bars represent 75 μm

We identified 7 mito-piRNAs expressed in all the cell types analyzed, including the 3T3-L1 NGS libraries (Fig. 5a). Selected mito-piRNAs were transcribed from mitochondrial non-coding genes (rRNA and tRNA) and from the non-coding D-loop region (Additional file 2: Table S1). We quantified selected mito-piRNAs by RT-qPCR (Fig. 5b) and the most strongly expressed piRNAs in our RT-qPCR experiments corresponded to piR-mmu-16,137,755 and piR-mmu-7,456,245, derived from the mt-TrnV and D-loop region, respectively (Fig. 5b). By contrast, the piR-mmu-775,559 and piR-mmu-9,238,634 derived from the ribosomal mitochondrial gene, were not detected (Fig. 5b) by RT-qPCR, consistent with their weaker expression in NGS libraries (Fig. 5a).

For confocal analyses and cell respiration assays, we selected *piR-mmu-7,456,245* for two reasons: 1) its relatively high expression in all cell types; 2) because is encoded in the D-loop region, a key regulatory region of mitochondrial genome [8, 9] (Fig. 1, piRNA circle, black arrow). As mito-piR-7,456,245 was a good candidate to explore its potential function in mitochondria, specific anti-piRNA LNA™ GapmeR probes were designed based on the *mito-piR-7,456,245* sequences (Additional file 2: Table S1) and AntipiRNA-F was also used to detect the *mito-piR-7,456,245* in the mitochondria of 3T3-L1 cells (Fig. 5c). The punctuate cytoplasmic AntipiRNA-F signal detected indicated that the gymnotic method of transfection allowed these oligonucleotides to enter the cells (Fig. 5d). To assess the role of *mito-piR-mmu-7,456,245* in mitochondria, we attempted to inhibit its regulatory activity using an Anti-piRNA LNA™ GapmeR probe, analyzing the bioenergetic properties of the 3T3-L1 cell line. We were unable to detect any significant differences between the cell lines transfected with either the specific Anti-piRNA or the scramble negative probe A (SRC-A) (Additional file 1: Figure S6) under different experimental conditions. This failure to alter the respiratory capacity of the cells was indicative of the presence of a fully functional mitochondrial mass, although it may reflect the inability of this probe to access the mitochondrial matrix or weak RNase H activity in the mitochondria.

## Discussion

In addition to serving as the powerhouse of cells, mitochondria play a key role in maintaining cellular homeostasis [3]. Indeed, mitochondria fulfill a pivotal role in reproduction, both in the development and maintenance of germ cells and gametes, as well as in successful fertilization and early embryonic development [30]. The discovery of sncRNAs encoded by mtDNA, called mitosRNAs [15], opens the door to novel mechanisms of regulation in mitochondria and of their interactions with other cellular components [17].

Here, we analyzed all the mitosRNAs encoded by the mitochondria of male mouse germ cells, gametes and zygotes, classifying them into different functional groups of sncRNAs [15]. If these were non-specific RNA turnover products, one would expect a random distribution of RNA sizes, no nucleotide preference at either end or non-specific cell expression. However, while there was significant diversity among the mitosRNAs, they also displayed cell-specific expression. Our bioinformatics workflow allowed us to classify mitosRNA into functional sncRNA populations. As such, piRNAs were the most abundant sncRNA population in the mitochondria of the mouse cells analyzed. Our bioinformatics approach also yields additional information of interest regarding these new mitochondrial sncRNAs.

To date, miRNAs are the best studied sncRNAs in the mitochondria [17, 26], although we also identified miRNA sequences encoded by mtDNA and these mito-miRNAs represent a large proportion of the miRNAs isoforms detected. In addition, it is known that some isomiRs can act in conjunction with canonical isoforms to target functionally related genes [31] and thus, we hypothesize that these isoforms could cooperate/interfere with mature miRNAs in the cytoplasm. However, it will be necessary to obtain more data to establish the true role of these isoforms, even in the case of cytosolic miRNAs, as they too are still poorly understood. In an attempt to gain some insight into this issue, we performed an in silico target analyses of the *mito-miR-6390* isomiR expressed in all the cell types analyzed. This functional analyses of the isomiR-6390 target genes suggested possible roles in the regulation of fatty acid metabolism via the PPAR signal pathway. Recent studies showed that nuclear *miR-199a-5p* and *miR-29a-3p* are present in mitochondria, and that they also regulate mitochondria metabolism via PPAR pathway [26]. The possibility that mitochondria may modulate gene expression using miRNAs opens the door to a new level of communication between the mitochondria and their cellular milieu.

The PIWI biogenetic pathway is associated to mitochondria organelle [32] and it participates in the formation of a key developmental structure in *D. melanogaster* germ cells, the “nuage” [29]. However, only one study to our knowledge has focused on the piRNAs encoded by mtDNA [21]. In mammalian cancer cells, mito-piRNAs are mainly encoded by non-coding mt-rRNAs and mt-tRNAs genes, with asymmetric mt-RNA fragment usage and cell type specific expression [21]. The mito-piRNAs detected through our in silico approaches in male germ cells, gametes and zygotes present similar characteristics to the mito-piRNAs previously identified in mammalian cells [21]. In addition, our in silico analyses detected the expression of mito-piRNAs encoded in the non-coding region, such as the D-loop. It should be noted that there is differential production of mito-piRNAs from this region, mainly in SPZs that do not produce the same mito-piRNAs as male germ cells or female gametes. Indeed, we confirmed the expression of 7 mito-piRNA sequences by RT-qPCR in 3T3-L1 cells.

Finally, the *mito-piR-7,456,245* sequence was present in the mitochondria of 3T3-L1 cells and we focused on this mito-piRNA as it is expressed from the D-loop region, an important non-coding regulatory region [8, 9]. The *mito-piR-7,456,245* sequence was found up-regulated in the hippocampus of trained mice [33] and it is one of the most strongly expressed piRNAs in the D-loop region in all cell types. For these reasons, we used an antisense LNA™ GapmeR to target *piR-7,456,245* and shed light on the role of this piRNA in mitochondrial metabolism. However, we did not detect any effect of *piR-7,456,245* on mitochondrial respiration, although it is not clear if the LNA GapmeR targets sncRNAs inside the mitochondria. Further studies with optimized RNA inhibitors will be necessary to address the role of mito-piRNAs in mitochondria. Nevertheless, this is one of the first studies to characterize piRNAs encoded by mtDNA in mammalian cells [21].

The mitochondrial nuclear genome is the result of the transfer of mtDNA fragments during evolution, including those in the D-loop region [34, 35]. These nuclear sequences of mitochondrial origin (called NUMTS) [23] retain repetitive elements, and they can intrinsically house TEs [24, 36]. The presence of relevant mito-piRNAs is consistent with the germline nature of the cells analyzed and the high proportion of the mitosRNAs associated with the NUMTS identified. However, the existence and activity of TEs in the mtDNA is more controversial. Some TE-like sequences have been considered to be present in yeast and plant mtDNA, yet more detailed analyses indicate that these sequences arise in the mtDNA as fragments from nuclear-derived TE insertions [37, 38]. Activation of TEs in mtDNA does not appear to be common in animals and consequently, the stability of the mtDNA with respect to transposition activity is high. Therefore, it is tempting to speculate that the mito-piRNAs derived from mtDNA might be involved in functions other than the classic repression of TEs in germ cells, as we proposed previously [18, 20, 21]. Together, the data presented here lead us to hypothesize that piRNAs, as well as their associated proteins, play a key role in mitochondrial homeostasis and nuclear communication. Recent studies of small mitochondrial highly-transcribed RNAs (smithRNAs) in *Ruditapes philippinarum* (Mollusca Bivalvia) gonads indicated that these RNAs could be involved in gonad development, influencing nuclear gene expression [39]. This hypothesis should be studied in more depth, since mitosRNAs (or Bivalvia smithRNAs) could participate in the important communication that develops between the mitochondria and nucleus [17, 39].

## Conclusions

Here we describe the mitoRNA landscape encoded by mouse male germ cells, gametes and zygotes. Our data suggest that these sncRNAs have regulatory functions in mitochondria that are related to the activity of gametes and of zygotic cells, each of which display specific patterns of expression and a wide diversity of mitosRNAs. We have also discovered that mito-piRNAs are the most abundant population of mitosRNAs, although their function and biogenesis in mitochondria is still poorly understood. Nevertheless, the identification of mito-piRNAs in different mammalian cell types opens the door to new studies that explore the role of this revolutionary group of sncRNAs in mitochondria. In these regard, we think that it will be important to characterize the expression profiles and diversity of mitosRNAs in human oocytes, not least because they may be a vital element in successful mitochondrial replacement therapies.

## Methods

### Data collection

Following our earlier studies, small RNA-seq libraries from male mouse PGCs (obtained on day 13 post-conception, E13), spermatogonia, oocytes, spermatozoa, zygotes and 3T3-L1 cells were recovered from a GenBank RSA archive (accession numbers SRX648519–23, SRR1269676–7 and SRR390297, respectively). The 3T3-L1 reads were collapsed in a unique fastq file and the average read count was taken for the expression analysis. Small RNA-seq libraries were generated and the adapters trimmed as described previously [19, 22]. The trimming reads were collapsed and sequences with five counts or less were removed from the study to reduce potentially inconsistent functional sequences.

### Bioinformatics workflow

After trimming and cleaning the reads, a bioinformatics analysis was performed according to the pipeline described in Additional file 1: Figure S1. The mouse nuclear genome (mm10) was downloaded from the Ensembl database and the “gold” mitochondrial genome (AY172335) from the NCBI database. All the mapping steps in the bioinformatics pipeline were performed using the Bowtie aligner (http://bowtie-bio.sourceforge.net/index.shtml). The read counts were obtained using HTSeq-count [40] and they were normalized using the DESeq package (http://bioconductor.org/packages/release/bioc/html/DESeq.html).

### In silico identification and classification of sncRNAs

The classification of sncRNAs associated with mitochondria in the different databases was performed by sequential mapping using Bowtie, as described in Additional file 1: Figure S1. Briefly, miRNAs were identified using mirBASE v21 (http://www.mirbase.org/) and isomiRs (see below) were classified using the scripts described elsewhere [13, 41]. We used an immunoprecipitation piRNA database for piRNA identification that we assembled previously (IPpiRNA db, https://github.com/edugenetico/Immunoprecipitation-piRNA-database.git) and that is derived from piRBase (http://regulatoryrna.org/database/piRNA/). Other types of ncRNAs were identified using Emsembl ncRNA sequences (http://www.ensembl.org/info/data/ftp/index.html). Reads that were not mapped in these databases were classified as unannotated. The mitochondrial read coverage was estimated using the BEDTools suite (http://bedtools.readthedocs.io/en/latest/content/bedtools-suite.html), applying the *default parameters. To determine the coverage of the 5′ and 3’ tRNA arm, the average read depth for each fragment was calculated based on the 5′ and 3′ region defined by the anticodon in each mitochondrial tRNA.*

### Additional software and analyses

*Heatmaps and* hierarchical clustering *were performed using the MeV program (*https://sourceforge.net/projects/mev-tm4/*). C*ircular plots of the mitochondrial genome associated sncRNAs were created using coverage data from BEDTools and Circleator v1.o (http://jonathancrabtree.github.io/Circleator/), and gene annotation and classification was retrieved from Mouse Genome Informatics (MGI, http://www.informatics.jax.org**/).** For miRNA target prediction, the information regarding miRNA:target interactions in the mirDB was used **(**http://www.mirdb.org**/)** with a threshold of 0.6, as well as that from the DIANA-microT web server v5 [42] with a threshold of 0.7. The targets predicted by the two web tools were subjected to functional annotation using DAVID (https://david.ncifcrf.gov**/**).

### 3T3-L1 cell culture and RNA isolation

3T3-L1 mouse embryo fibroblasts (obtained from the American Type Culture Collection, Mansassas, VA) were cultured at 37 °C in a humidified atmosphere of 5% CO_2_ in Dulbecco’s modified Eagle’s medium (DMEM) containing 10% (*v*/v) heat-inactivated bovine serum (FBS) supplemented with penicillin (100 U/ml) and streptomycin (100 μg/ml**).** To recover the cells when confluent (80–70% cells density), they were digested with 0.05% Trypsin-EDTA for 3 min at 37 °C and isolated from the culture medium by centrifugation. Total RNA was isolated from 3T3-L1 cells using the NZYol reagent (NZYTech) and DirectZol (Zymo Research), according to the manufacturer’s instructions. RNA quantification and integrity was evaluated on a 2100 Bioanalyzer (*Agilent Technologies).*

### Quantitative RT-PCR of piRNAs in 3T3-L1 cells

Reverse transcription of sncRNAs was performed using 1 μg of total RNA isolated from 3T3-L1 cells, the miScript II RT Kit (Qiagen) and HiFlex Buffer, according to the manufacturer’s protocol. The expression of specific piRNAs was assessed by quantitative RT-PCR (RT-qPCR) using the miScript SYBR Green PCR Kit (Qiagen), a Universal miScript Primer (Qiagen) and specific primers for each piRNA (Additional file 2: Table S1). The U6 derived piRNA (piR-mmu-49,487,030) was used as an endogenous control for RT-qPCR and all the reactions were performed in a *LightCycler 480* System (Roche), carrying out primer efficiency and melting analyses for all the qPCR primers.

### Confocal image visualization

Antisense LNA™ GapmeR (AntipiRNA) and fluorescein labelled AntipiRNA (AntipiRNA-F) were designed by Exiqon, based on the piR-mmu-7,456,245 sequence (Additional file 2: Table S1). A negative control (SRC-A: Antisense LNA GapmeR, negative control A) was also purchased from Exiqon. The 3T3-L1 cells were cultured on sterile *cover*slips and antipiRNA-F was added to the medium at a final concentration of 100 mM using gymnotically delivery of LNA GapmeR [43].

### 3T3-L1 cell mitochondrial respiration

The XF24 Extracellular Flux Analyzer (Seahorse Biosciences) was used to determine the bioenergetics profile of 3T3-L1 cells after transfection with SRC-A, AntipiRNA-F and AntipiRNA LNA GapmeR. Briefly, 10^3^ cells per well were seeded in XF24 plates (Seahorse Biosciences) and allowed to recover for 24 h. The cells were then transfected by gymnosis [43], maintained for 48–72 h with AntipiRNAs concentrations ranging from 50 to 250 nM and subsequently, the medium was changed to bicarbonate-free DMEM (Sigma-Aldrich) supplemented with 11.11 mM glucose, 2 mM L-glutamine, 1 mM pyruvate, and 2% FBS (Sigma-Aldrich) and they were maintained for 60 min in a CO_2_-free incubator. The oxygen consumption rate (OCR) and extracellular acidification rate (ECAR, a proxy for lactate production) of the cells were recorded to assess the mitochondrial respiratory and glycolytic activity, respectively. After taking four measurements under basal conditions, the cells were treated sequentially with 1 μM oligomycin, 0.6 μM or 0.4 μM carbonyl cyanide *p*-[trifluoromethoxy]-phenyl-hydrazone (FCCP), and 0.5 μM rotenone plus 0.5 μM antimycin A, taking three consecutive measurements under each condition. Non-mitochondrial respiration (the OCR value after addition of rotenone plus antimycin A) was subtracted from all the OCR measurements and the total protein concentration was determined with the BCA assay using albumin as a standard.

## Additional files

Additional file 1: **Figure S1.** Bioinformatics pipeline for the sncRNAs derived from mtDNA. **Figure S2.** Density of the sncRNA reads per Kb. DNA from the mouse nuclear genome and mtDNA. **Figure S3.** Nuclear mitochondrial sequences (NUMTs) identified in the mouse genome. **Figure S4.** Average nucleotide coverage of the mito-piRNAs in mt-tRNAs. **Figure S5.** Characterization of mitosRNA from the 3T3-L1 cell datasets. **Figure S6.** Effects of mito-piR-7,456,245 inhibition on mitochondrial respiration in 3T3-L1 cells. (PDF 125 kb)

Additional file 2: **Table S1.** List of piRNA-specific primer sequences used for qPCR. **Table S2.** List of targets for *mmu-miR-6390* predicted by miRDB and DIANA-microT. **Table S3.** In silico analysis of the mitochondrial localization of mouse PIWI protein isoforms. **Table S4.** Description of the small RNA-Seq datasets in 3T3-L1 cells obtained from the GEO database. (PDF 1076 kb)

## Abbreviations

endo-siRNAs: Endogenous-small interfering RNA
mitomiR: Mitochondrial miRNA
mitosRNA: Mitochondrial-associated RNA
ncRNA: Non-coding RNA
NUMTs: Pecific nuclear mitochondria sequences
OCY: Mouse oocytes
PGC: Mouse primordial germ cells at 13dpc
piRNAs: Piwi-interacting RNA
RISC: RNA-induced silencing complex
SPG: Mouse spermatogonia cells
SPZ: Mouse spermatozoa
ZYGO: Mouse zygotes
