# Supplementary material for: The landscape of mitochondrial small non-coding RNAs in the PGCs of male mice, spermatogonia, gametes and in zygotes
Source: BMC Genomics. 2018 Aug 28;19:634. doi: 10.1186/s12864-018-5020-3 (PMC6114042; doi:10.1186/s12864-018-5020-3)
Supplement: Supplementary file 1 — Figure S1. Bioinformatics pipeline for the sncRNAs derived from mtDNA. Figure S2. Density of the sncRNA reads per Kb. DNA from the mouse nuclear genome and mtDNA. Figure S3. Nuclear mitochondrial sequences (NUMTs) identified in the mouse genome. Figure S4. Average nucleotide coverage of the mito-piRNAs in mt-tRNAs. Figure S5. Characterization of mitosRNA from the 3T3-L1 cell datasets. Figure S6. Effects of mito-piR-7,456,245 inhibition on mitochondrial respiration in 3T3-L1 cells. (PDF 125 kb) [file 12864_2018_5020_MOESM1_ESM.pdf]

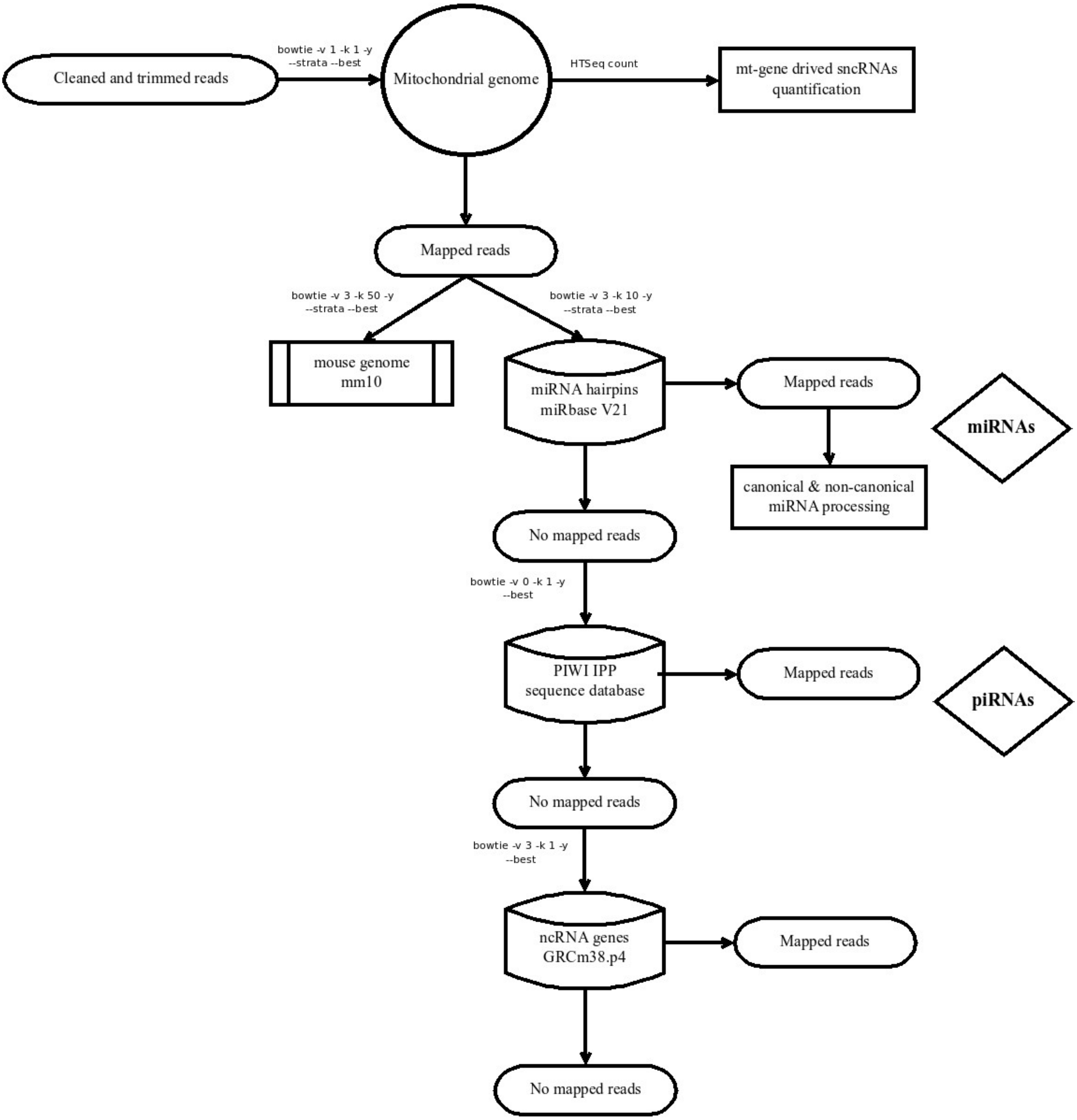

Figure S1. Bioinformatics pipeline for the sncRNAs derived from mtDNA

Schematic diagram of sncRNA-seq processing. Mapping program and parameters were indicated. Bowtie (http://bowtie-bio.sourceforge.net/manual.shtml) and HTSeq count (http://www.huber.embl.de/users/anders/HTSeq/doc/overview.html). Databases: miRBase V21 (http://www.mirbase.org/), PIWI IPP (https://github.com/edugenetico/Immunoprecipitation-piRNA-database.git) and ncRNA genes from (http://www.ensembl.org/info/data/ftp/index.html).

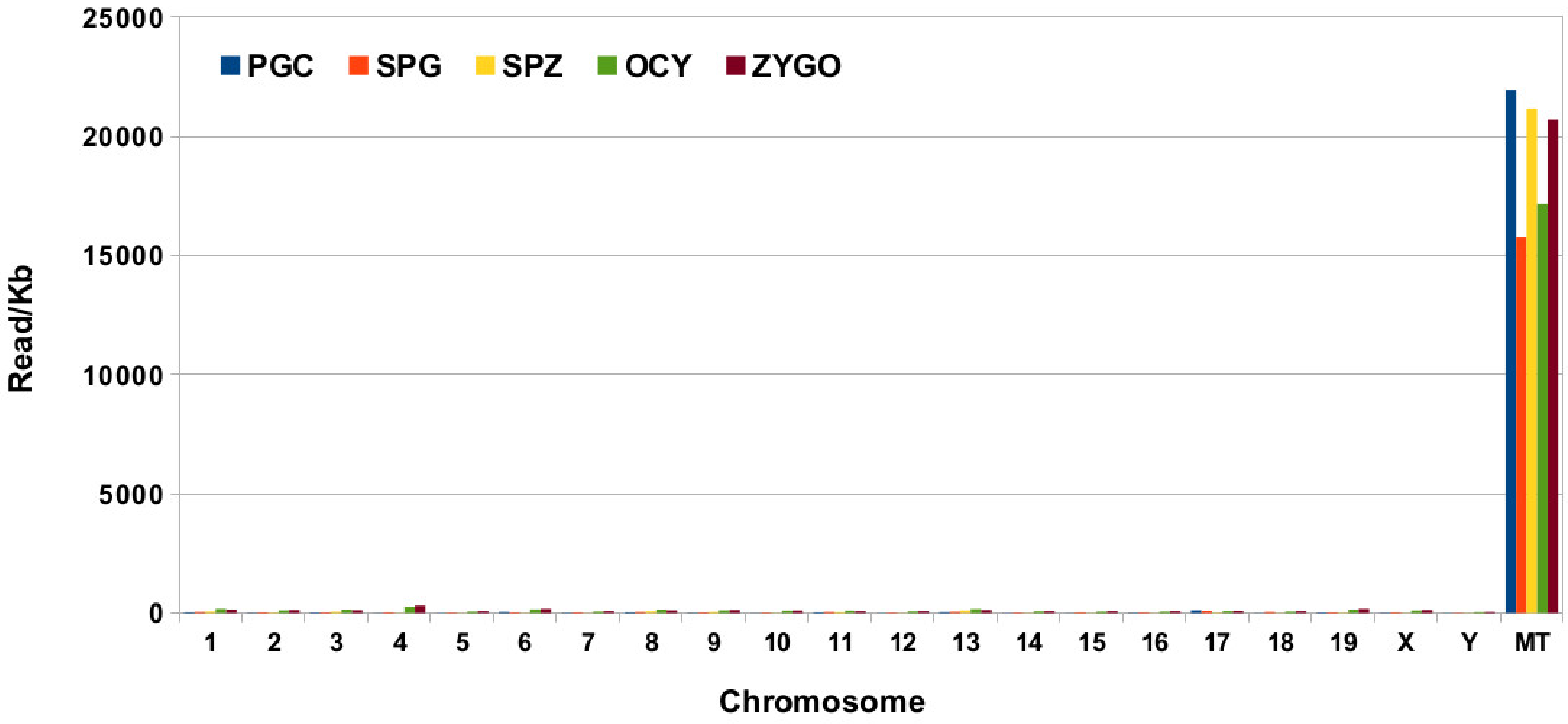

Figure S2. Density of the sncRNA reads per Kb DNA from the mouse nuclear genome and mtDNA.

Production of sncRNAs reads from the different chromosomes was carried out using the sncRNA clean reads from PGCs, spermatogonia, spermatozoa, oocytes, and zygotes small RNA-seq libraries. Clean reads were mapped against the mm10 mouse genome containing the mouse mitochondria genome. Reads were aligned using Bowtie up to 1 mismatches and the best ten alignments. Reads per Kb was calculated dividing the mouse chromosome size by the number of mapping reads present in each chromosome.

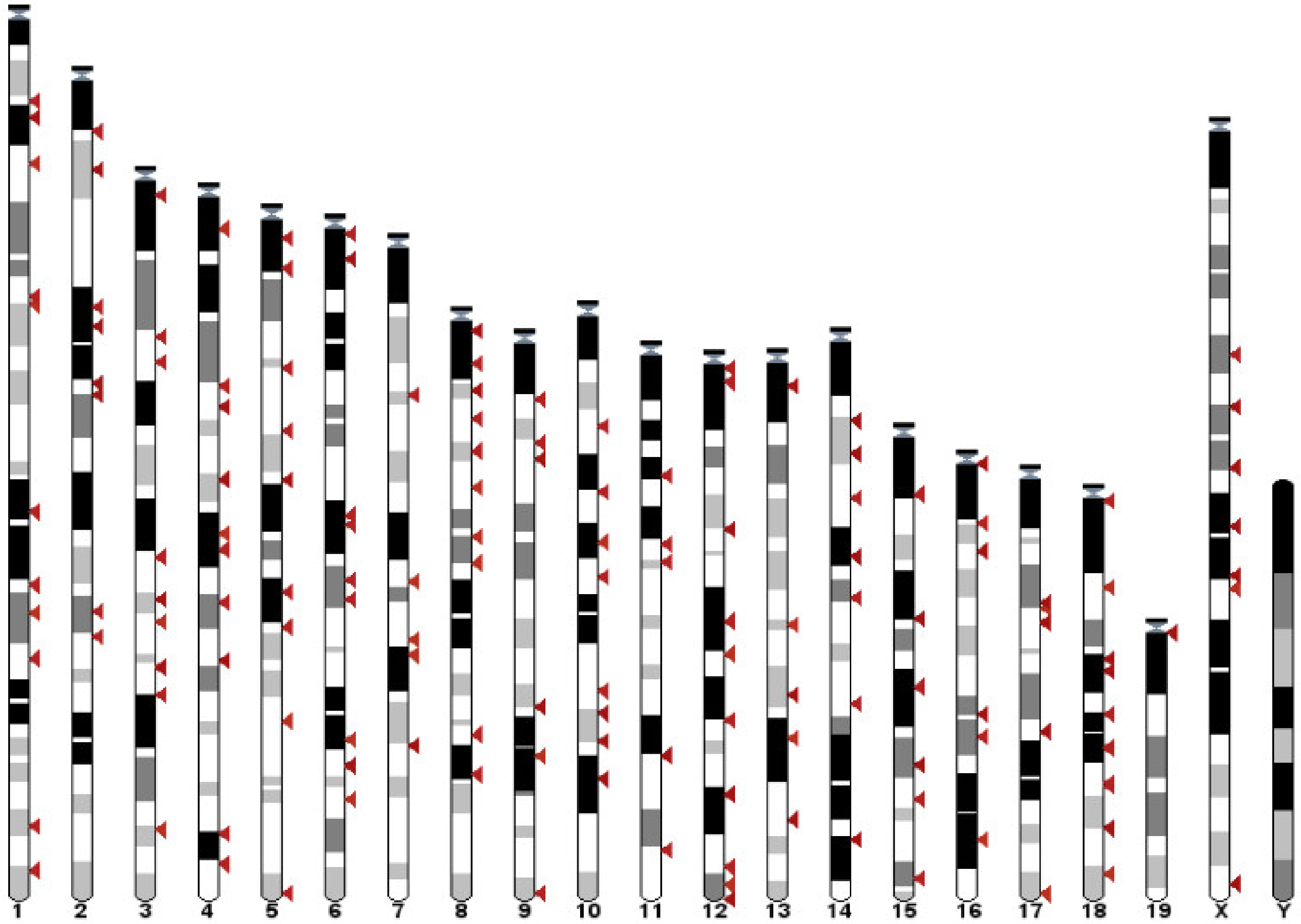

Figure S3. Nuclear mitochondrial sequences (NUMTs) identified in the mouse genome.

Identification of NUNTs in mouse genome was carried out by Blast search in Ensembl database. Sequences from coding and non-coding genes of mitochondrial genome were obtained from Emsembl, and used as a query for Blastn analyses against the mm10 mouse genome assembling. Image shows a mitocondrial sequences (red triangles) distribution in mouse chromosomes.

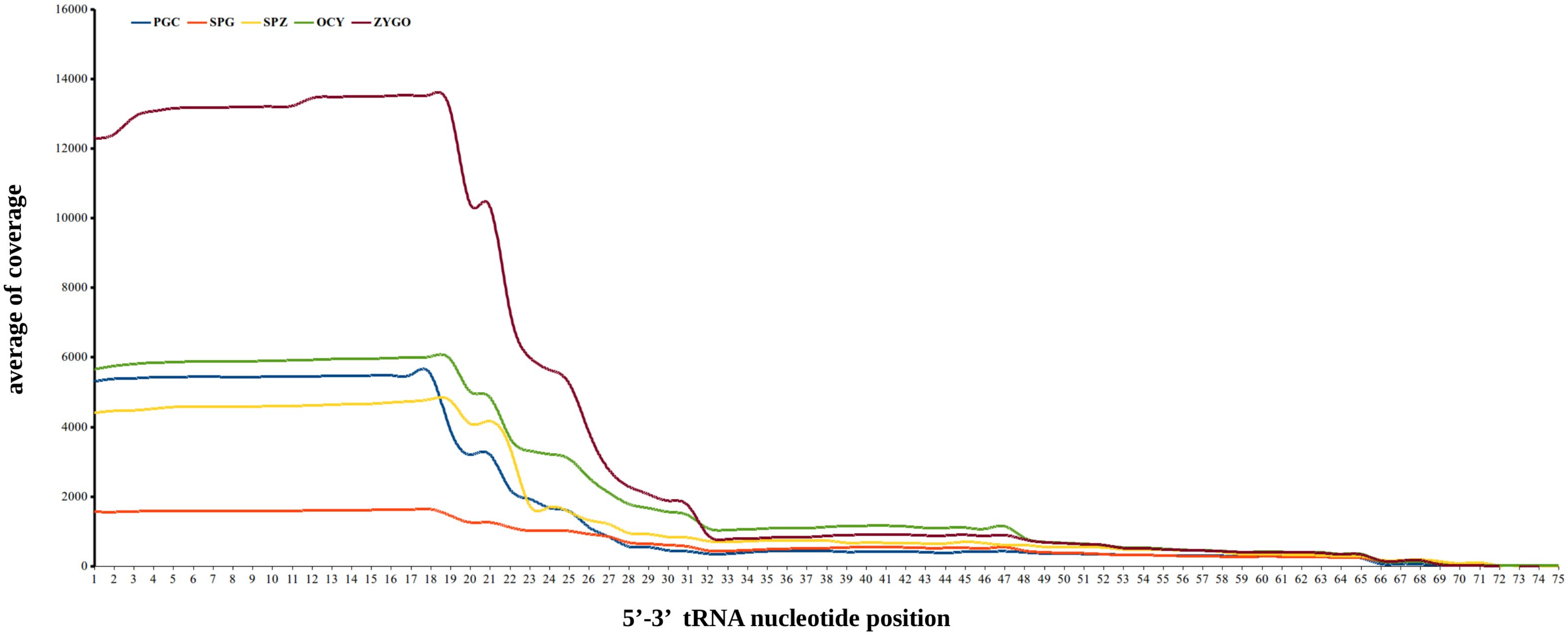

Figure S4. Average nucleotide coverage of the mito-piRNAs in mt-tRNAs.

Average of coverage per nucleotide position of mitopiRNAs in mt-tRNAs. Per base coverage was calculated using BEDTools suite (http://bedtools.readthedocs.io/en/latest/content/bedtools-suite.html). Different cell types are represented by different colors. Graphs represent the average of coverage of mitopiRNA in different mt-tRNA. Data was not normalized.

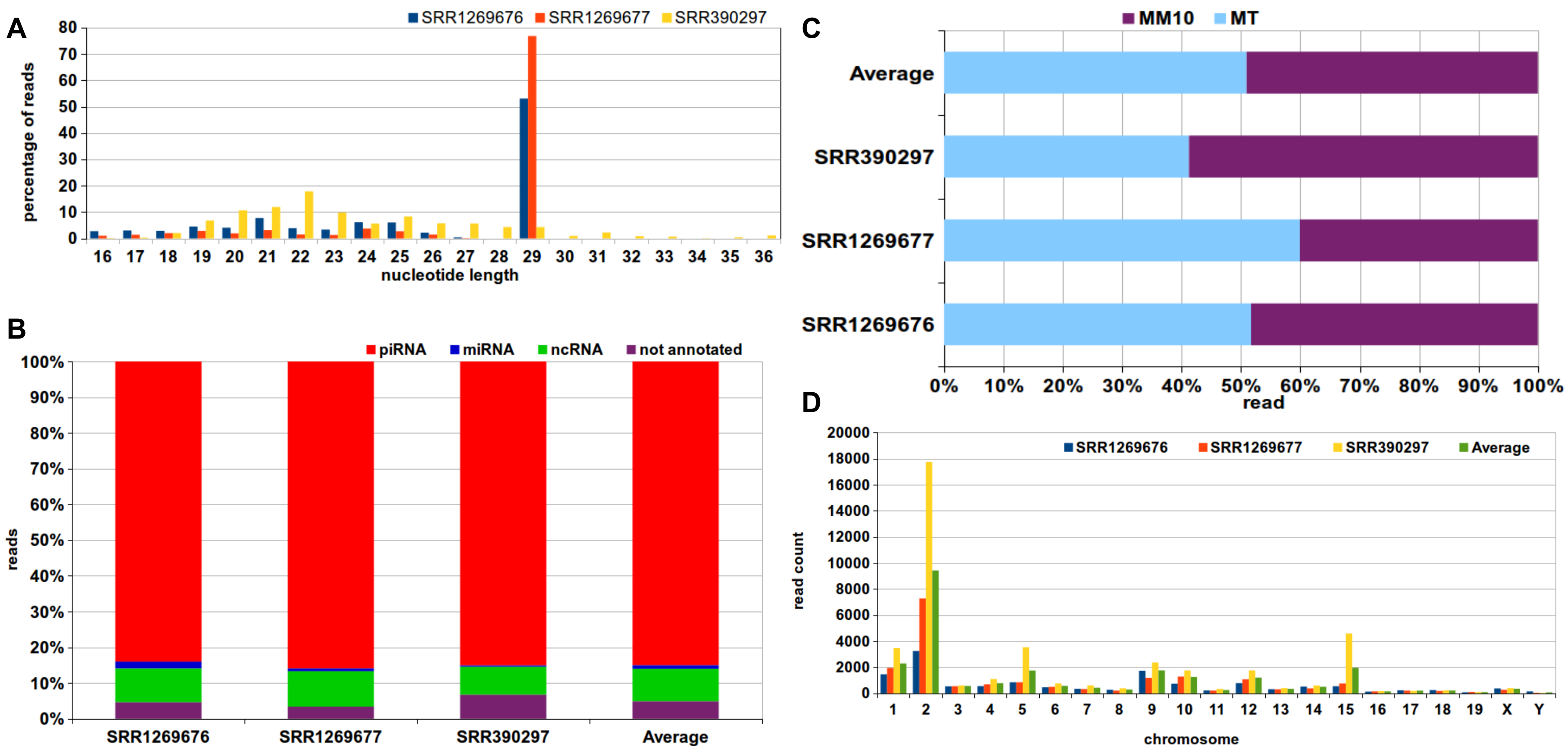

Figure S5. Characterization of mitosRNA from the 3T3-L1 cell datasets

A) Read length distribution of sncRNAs derived from mitochondria of 3T3 dataset from GEO database. Percentage of reads was calculated from total reads present in the small RNA-Seq library. B) Classification of 3T3 mitochondria encoded sncRNAs in microRNAs (miRNA, blue), PIWI interacting RNA (piRNA, red) and sequences from non-coding RNAs present in Ensembl database (ncRNAs, green). Reads that not map in previous databases are considered as not annotated in purple. Average correspond to mean of 3 NGS libraries read counts . C) Percentage of mitochondrial encoded sncRNAs that map in mouse genome (MM10) and exclusives of mitochondrial genome (MT). Average correspond to mean of 3 NGS libraries D) Mouse chromosome distribution of read from identified mitochondria associated sncRNAs. Average correspond to mean of 3 NGS libraries

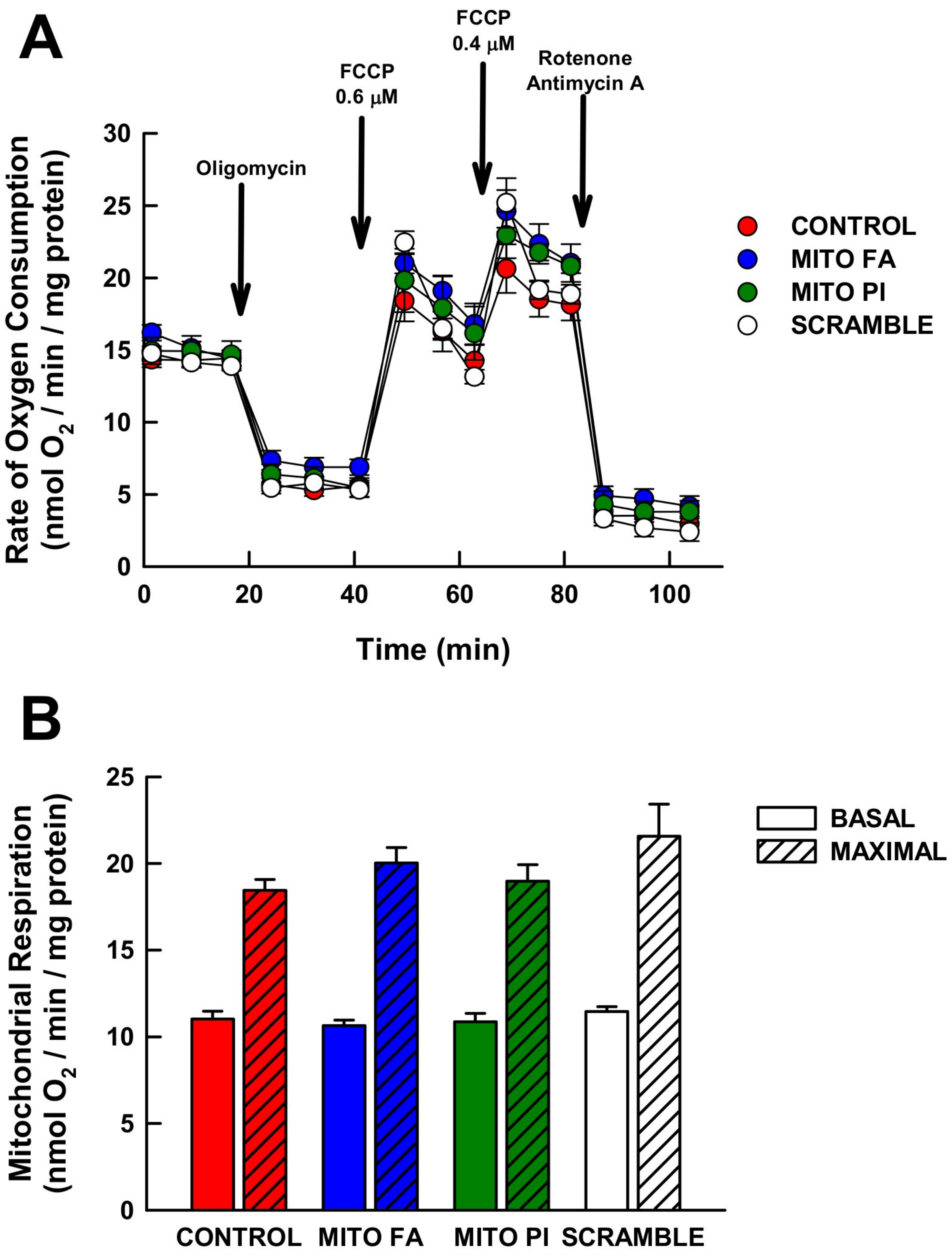

Figure S6. Effects of mito-piR-7456245 inhibition on mitochondrial respiration in 3T3-L1 cells.

A) Cellular metabolism characterization of 3T3-L1 cells 48 hours after transfection with 250 nM specific *piR-7456245* antisense LNA GapmeR, 250 nM (MITO PI) and *piR-7456245* antisense LNA GapmeR fluorescein label (MITO FA) or 250 nM scramble negative probe (SCRAMBLE) and in non-transfected control cells. The characterization was performed using the Seahorse XF24 extracellular flux analyzer. Oligomycin, FCCP and rotenone/antimycin A were added as indicated in the figure to determine the different bioenergetic parameters. B) Summary of the effects of *piR-7456245* inhibition on the basal and maximal respiration of 3T3-L1 cells.
