# Supplementary material for: The landscape of mitochondrial small non-coding RNAs in the PGCs of male mice, spermatogonia, gametes and in zygotes
Source: BMC Genomics. 2018 Aug 28;19:634. doi: 10.1186/s12864-018-5020-3 (PMC6114042; doi:10.1186/s12864-018-5020-3)
Supplement: Supplementary file 2 — Table S1. List of piRNA-specific primer sequences used for qPCR. Table S2. List of targets for mmu-miR-6390 predicted by miRDB and DIANA-microT. Table S3. In silico analysis of the mitochondrial localization of mouse PIWI protein isoforms. Table S4. Description of the small RNA-Seq datasets in 3T3-L1 cells obtained from the GEO database. (PDF 1076 kb) [file 12864_2018_5020_MOESM2_ESM.pdf]

Supplementary Tables

Table S1. List of piRNA-specific primer sequences used for qPCR

PirBase ID	Primer sequence	Associated gene
piR-mmu-5890709	5'-ACTAATCCTAGCCCTAGCCCTACA-3'	Rnr2
piR-mmu-16137755	5'-CATAGTGTTGCTTAATATTAAAGCATCTG-3'	TrnV
piR-mmu-9238634	5'-AACATAAAAACGTTAGGTCAAGGTGTAGC-3'	Rnr1
piR-mmu-25691968	5'-GTTAATGTAGCTTAATAACAAAGCAAAGC-3'	TrnF
piR-mmu-5850420	5'-CAAGAAGTAGTTTAATTAGAATACCAGCTT-3'	TrnP
piR-mmu-20317834	5'-ATTCACAGTAGGATTAGATGTAGACAC-3'	COX I
piR-mmu-775559	5'-CAGACTATAGGCAATAATCACACTATAAA-3'	Rnr2
piR-mmu-5142078	5'-ACTAATCCTAGCCCTAGCCCTACAC-3'	Rnr2
piR-mmu-49487030	5'-GCAAATTCGTGAAGCGTTCC-3'	U6
piR-mmu-7456245	5’-GAATTGATCAGGACATAGGGTTTGATA-3'	D-Loop

Table S2. List of targets for *mmu-miR-6390* predicted by miRDB and DIANA-microT

Ensembl Gene ID	Gene	Annotation	miTG	TPS
ENSMUSG00000026499	Acbd3	acyl-Coenzyme A binding domain containing 3	0.9	95
ENSMUSG00000021076	Actr10	ARP10 actin-related protein 10	0.8	70
ENSMUSG00000024654	Asrgl1	asparaginase like 1	0.8	81
ENSMUSG00000029467	Atp2a2	ATPase, Ca++ transporting, cardiac muscle, slow twitch 2	0.9	96
ENSMUSG00000069895	Atxn1l	ataxin 1-like	0.7	85
ENSMUSG00000050996	Cetn1	centrin 1	0.8	76
ENSMUSG00000031327	Chic1	cysteine-rich hydrophobic domain 1	0.8	68
ENSMUSG00000039652	Cpeb3	cytoplasmic polyadenylation element binding protein 3	0.8	66
ENSMUSG00000026385	Dbi	diazepam binding inhibitor	0.9	94
ENSMUSG00000039768	Dnajc11	DnaJ (Hsp40) homolog, subfamily C, member 11	0.7	81
ENSMUSG00000038174	Fam126b	family with sequence similarity 126, member B	0.7	82
ENSMUSG00000072568	Fam84b	family with sequence similarity 84, member B	0.9	54
ENSMUSG00000026193	Fn1	fibronectin 1	0.7	89
ENSMUSG00000039286	Fndc3b	fibronectin type III domain containing 3B	0.8	59
ENSMUSG00000029563	Foxp2	forkhead box P2	0.9	58
ENSMUSG00000051455	Gm1564	predicted gene 1564	0.8	91
ENSMUSG00000056870	Gulp1	GULP, engulfment adaptor PTB domain containing 1	0.8	59
ENSMUSG00000032293	Ireb2	iron responsive element binding protein 2	0.7	67
ENSMUSG00000047976	Kcna1	Ka voltage-gated channel, shaker-related subfamily, member 1	0.8	52
ENSMUSG00000022342	Kcnv1	potassium channel, subfamily V, member 1	0.7	80
ENSMUSG00000038056	Kmt2c	lysine (K)-specific methyltransferase 2C	0.7	56
ENSMUSG00000022139	Mbnl2	muscleblind-like 2	0.8	75

Table S2 (continuation)

Ensembl Gene ID	Gene	Annotation	miTG	TPS
ENSMUSG00000041132	N4bp2l1	NEDD4 binding protein 2-like 1	0.8	63
ENSMUSG00000040536	Necab1	N-terminal EF-hand calcium binding protein 1	0.7	72
ENSMUSG00000034701	Neurod1	neurogenic differentiation 1	0.9	73
ENSMUSG00000051118	Olfr77	olfactory receptor 77	0.9	70
ENSMUSG00000039361	Picalm	phosphatidylinositol binding clathrin assembly protein	0.7	76
ENSMUSG00000017754	Pltp	phospholipid transfer protein	0.8	68
ENSMUSG00000030704	Rab6a	RAB6A, member RAS oncogene family	1.0	66
ENSMUSG00000026970	Rbms1	RNA binding motif, single stranded interacting protein 1	1.0	61
ENSMUSG00000048027	Rgmb	repulsive guidance molecule family member B	0.7	62
ENSMUSG00000033282	Rpgrip1l	Rpgrip1-like	0.9	72
ENSMUSG00000051354	Samd3	sterile alpha motif domain containing 3	0.8	63
ENSMUSG00000023927	Satb1	special AT-rich sequence binding protein 1	0.7	51
ENSMUSG00000075316	Scn9a	sodium channel, voltage-gated, type IX, alpha	0.8	99
ENSMUSG00000044791	Setd2	SET domain containing 2	0.9	70
ENSMUSG00000021432	Slc35b3	solute carrier family 35, member B3	0.7	67
ENSMUSG00000027109	Sp3	trans-acting transcription factor 3	0.8	67
ENSMUSG00000056296	Synpr	synaptoporin	0.8	67
ENSMUSG00000029287	Tgfbr3	transforming growth factor, beta receptor III	0.9	53
ENSMUSG00000013701	Timm23	translocase of inner mitochondrial membrane 23	0.7	63
ENSMUSG00000048022	Tmem229a	transmembrane protein 229A	0.9	52
ENSMUSG00000024614	Tmx3	thioredoxin-related transmembrane protein 3	0.7	97
ENSMUSG00000033454	Zbtb1	zinc finger and BTB domain containing 1	0.9	89
ENSMUSG00000032846	Zswim6	zinc finger SWIM-type containing 6	0.8	82
ENSMUSG00000034430	Zxdc	ZXD family zinc finger C	0.8	86

Prediction scores: TPS, target prediction scores. Higher score, more confidence the prediction. 0-100. miRDB ; miTG, prediction score. The higher miTG score, higher probability of targeting. 0-1. DIANA-microT.

Table S3. ***In silico*** **analysis of the mitochondrial localization of mouse PIWI protein isoforms.**

				MitoProt II	TargetP 1.1	
Gene Name	Transcript name	Transcript ID	Protein	Probability	mTP	RC
MIWI	Piwil1-001	ENSMUST00000086056.7	862 aa	0.3534	0.762	3
	Piwil1-003	ENSMUST00000195959.1	835 aa	0.1393	0.762	3
	Piwil1-002	ENSMUST00000200192.4	493 aa	0.4362	0.593	5
MILI	Piwil2-201	ENSMUST00000048129.4	971 aa	0.4741	0.355	5
MIWI2	**Piwil4-201**	**ENSMUST00000076946.3**	**878 aa**	**0.9894**	**0.892**	**2**
	Piwil4-001	ENSMUST00000115644.8	848 aa	0.2982	0.867	2
	Piwil4-003	ENSMUST00000136399.7	174 aa	0.0529	0.501	5

Sequences, transcript name and ID from mouse PIWIs isoforms were obtained from Ensembl database (htttp://www.ensembl.org/index.html). Mitochondrial targeting and subcellular location of PIWI isoforms were carried out using MITOPROT II and TargetP 1.1, respectively. MitoProt probability to export protein to mitochondria,from 0-1 values. TargetP: mTP values represent a mitochondrial localization score, from 0-1 values; and reliability class (RC), from 1 to 5, where 1 indicates the strongest prediction.

MITOPROT II https://ihg.gsf.de/ihg/mitoprot.html

TargetP 1.1 http://www.cbs.dtu.dk/services/TargetP/

Table S4. Description of the small RNA-Seq datasets in 3T3-L1 cells obtained from the GEO database.

**NCBI RUN**	SRR1269676	SRR1269677	SRR390297
**GEO number**	GSE57138	GSE57138	GSE34475
**BioProject**	PRJNA246123	PRJNA246123	PRJNA151355
**Run**	SRR1269676	SRR1269677	SRR390297
**Reads**	5,401,690	5,946,592	16,537,853
**Reads cliping**	5,104,033	5,350,959	12,416,227
**reads map MT**	32,298	15,881	51,29
**% sncRNA MT**	0.6	0.3	0.4
